# Association Between Amerindian Ancestry and Chronic Obstructive Pulmonary Disease in the Chilean Mixed Population

**DOI:** 10.3390/jpm15040137

**Published:** 2025-03-31

**Authors:** Vicente Silva, Andrea Canals, Lucia Cifuentes

**Affiliations:** 1 Faculty of Medicine, University of Chile, Santiago 8380453, Chile; vicente.silva.a@ug.uchile.cl; 2 Biostatistics Program, School of Public Health, Faculty of Medicine, Santiago 8380453, Chile; acanals@uchile.cl; 3 Fundación Arturo López Pérez, Santiago 7500691, Chile; 4 Human Genetics Program, Institute of Biomedical Sciences, Faculty of Medicine, University of Chile, Santiago 8380453, Chile

**Keywords:** chronic obstructive pulmonary disease, ancestry, hospital discharge, Mapuche, Aymara, Amerindian, genetic susceptibility

## Abstract

**Background/Objectives**: Chronic Obstructive Pulmonary Disease (COPD) is one of the most common chronic non-communicable diseases in adults. The most critical risk factors are tobacco and air pollution. The familial aggregation of this disease and the fact that only 15–20% of smokers develop COPD demonstrate the existence of an individual susceptibility that would depend on genetic factors. The already-known susceptibility genomic variants explain only about 38% of the heritability of COPD. The present work analyzes the relationship between the percentage of Amerindian genomic ancestry of Chileans with morbidity and mortality of Chronic Obstructive Pulmonary Disease (COPD), adjusting for socioeconomic and environmental variables. **Methods:** We rely on the estimates of genomic ancestry percentages obtained in the Chilegenomico project in urban Chileans from 39 communes along eight regions of the country from north to south. From the public databases of the Departamento de Estadísticas e Información en Salud (DEIS) of the Chilean Ministry of Health, we obtained mortality rates and hospital discharge rates. We incorporated adjustment variables (communal data) obtained from other public databases. We performed correlation analyses and fitted negative binomial regression models to examine the association between Amerindian ancestries and COPD statistics. **Results:** A positive and significant association between Mapuche ancestry and hospital discharge and mortality rates for COPD was found in both simple and multiple analyses. In contrast, we found a negative and significant association between the percentage of Aymara genomic ancestry and COPD mortality rates. **Conclusions:** The levels of Mapuche and Aymara genomic ancestries have different and contrasting significant associations with COPD susceptibility and mortality in the Chilean mixed population.

## 1. Introduction

Chronic Obstructive Pulmonary Disease (COPD) is one of the most common chronic non-communicable diseases in adults, with prevalence increasing with age: it alone accounts for more than 3 million deaths per year worldwide [1]. In Chile, the prevalence is 16.9% in people over 40 years of age, is more frequent and severe in smokers [2], and results in a decrease in life expectancy [3]. COPD is also prevalent in other Latin American countries, especially in men and in smokers [4,5], with rates ranging from 7% in Mexico City to almost 20% in Montevideo [6]. The most critical risk factors are tobacco in high-income countries and air pollution in low-income countries [7,8]; other risk factors are occupational exposures and asthma [9]. The familial aggregation of this disease and the fact that only 15–20% of smokers develop COPD [10] demonstrate the existence of an individual susceptibility that would depend on genetic factors (polygenes) and their interaction with the environment. COPD is a disease of complex or multifactorial inheritance with an estimated heritability of about 60% [11]. It is characterized by an important chronic inflammatory component in the lung and airway in response to environmental pollutants [8,12]. Some aspects of its pathogenesis have been related to asthma and lung cancer. The clinical presentation is very heterogeneous, with different intensities of two essential components: emphysema and chronic limitation of the distal airway due to chronic inflammation and obstruction.

The first demonstration of a genetic factor directly linked to COPD was the finding that severe deficiency of alpha-1 antitrypsin (a plasma protease inhibitor of leucocyte elastase) [13], encoded by the SERPINA1 gene located on chromosome 14q32, significantly increases the risk of developing COPD. Severe deficiency of this enzyme explains between 1 and 3% of COPD cases in Caucasian populations. Subsequently, numerous GWAS (Genome-Wide Association Studies) have been carried out, demonstrating the role of several dozen genes in the development of COPD; these results have been replicated in several populations, although some differences have been observed between the results obtained in Asians and Europeans [14]. The genes identified in these studies are involved in the processes of morphogenesis during embryonic and fetal lung development, nicotine addiction, and tissue response to injury [15]. Among the latter are genes linked to the inflammatory response, protease-antiprotease balance, and antioxidant response [14].

In addition to the constitutional genomic variants, epigenetic mechanisms linked to COPD have been found [16,17,18,19], such as differential methylation of some genes, histone acetylation, and action of microRNAs [14]. A relationship between airway microbiome and disease development has also been proposed. Moreover, environmental factors like smoking and air pollution that interact with the genome to influence disease susceptibility have been widely described [7,8,15].

Several GWAS, replicated in different populations, have identified risk alleles in multiple genes, some with a significant effect and others a more moderate one [10]; most of them have been performed in Caucasian and Asian populations, a few in Hispanics from Costa Rica, Mexico, and the USA [20]. In South American populations, no GWAS have been performed for COPD; however, one study analyzed the association between 754,159 SNPs and COPD risk in 214 patients and 193 healthy controls from the Maule region in Chile and found risk SNPs in two genes not previously reported [21].

Despite all the efforts and risk alleles identified, known susceptibility genomic variants explain only about 38% of heritability [22], which leaves a significant proportion unexplained [17]. This suggests that there are still risk genes to be discovered, possibly alleles of low frequency in Caucasian and Asian populations, which could be more relevant in human populations not studied through GWAS, such as the populations of South America. Likewise, some of the genes identified in other populations may be less important in these unstudied human populations. The study by Díaz-Peña in a mixed Chilean population shows that the association of some alleles already known to be linked to COPD is only replicated in those Chileans with low Mapuche ancestry (<35%), but not in those with higher [21].

Ancestry refers to the information in our genetic repertoire that informs us about the geographic origin of our biological ancestors and the genetic links that unite us to them [23]. The ethnic origin of human populations, on the other hand, alludes to social characteristics that have cultural, racial, religious, and even political importance. Thus, in this work, we focus on the study of genomic ancestry, centered on the genetic resemblance that a mixed population, such as the Chilean population, has with the ancestral populations that gave rise to it.

Information on the percentage of different ancestries present at the genomic level in a mixed population can contribute to research on factors that influence the incidence and prevalence of diseases in different regions of a country, continent, or even the world. Finding a correlation between the percentages of genomic ancestry of a specific population and the susceptibility to certain diseases can help us to identify genetic risk factors for the disease under study by means of the admixture mapping method [24,25,26]; this method is useful in populations that are a result of a recent racial mixture like Chileans or Latin Americans in general. On the other hand, detecting this correlation, even if environmental and genetic factors cannot be distinguished, can contribute to defining prevention and treatment strategies that are better adjusted to the particular population.

To learn the ancestry of a mixed population, specific sectors or elements of the genome that vary between populations of different geographical origins must be studied; these must have as an essential characteristic a sufficiently different allelic frequency between the ancestral populations that gave rise to the current mixed population to be characterized. Important tools used in this type of study are the ancestry informative genetic markers (AIMs); of these, the most widely used are single nucleotide polymorphisms (SNPs) [27,28,29,30].

The average percentages of ancestry estimated in the Chilean urban population have been 44% Amerindian, 51% European, and 4% African, with regional variation: a higher percentage of Mapuche ancestry towards the south and a higher percentage of Amerindian ancestry, specially Aymara, towards the north [31,32]. We must briefly remember that the Chilean population was born mainly from a mixture that occurred from the 16th century onwards between the Spanish colonizers and the native peoples that had lived in our territory for thousands of years. Hence, the Chilean ancestry is mainly European and Amerindian [33,34]. The latter component comes from dozens of native peoples, two of whom are the most relevant: in the North (Aymara) and the center-south (Mapuche) of the country. The contemporary Chilean urban population is entirely mixed mestizos, and there are no pure original groups that can be categorically separated. Because of this, our study relied on admixed people with a continuous gradient of ancestry.

Previous studies have found relationships between a population’s ancestry and a disease’s epidemiology, such as the relationship between Mapuche ancestry and gallbladder cancer [35] and the association between European ancestry and breast cancer [36].

The present work aims to explore in the contemporary Chilean mixed population the association between the percentage of Amerindian ancestry (North Amerindians with high Aymara component and South Amerindians with high Mapuche component) with mortality rates and hospital discharges for COPD (as an approximation to the prevalence of the disease) in several communes (municipalities) of Chile that vary in the average percentage of Amerindian/European ancestry of their inhabitants. This could guide future research to explore genetic factors involved in COPD.

## 2. Materials and Methods

We used the estimates of ancestry percentages obtained in the framework of the Chilegenomico project, which includes information from inhabitants of 39 communes from 8 regions (I, IV, VII, VIII, IX, X, XIII, and XV), collected between 2012 and 2015. These estimates were made based on a panel of 147 informative ancestry SNPs [31,32] that provided information on the proportion of Amerindian, European, Aymara, and Mapuche ancestry present in the autosomal genome of Chileans (people born in Chile whose parents were also born in Chile) from the urban population.

We studied the susceptibility of Chileans to COPD using the hospital discharge rate as an indirect approximation to prevalence, and additionally, we analyzed the mortality rate. These data were obtained from the open-access databases of the Departamento de Estadísticas e Información en Salud (DEIS) of the Ministry of Health, Chile [37]. We expressed the hospital discharges and mortality rates per 100,000 inhabitants of the commune and extracted the population sizes of the different communes for 2015 and 2016 from the 2017 census database [38].

The necessary sample size to evaluate the association between ancestry (Amerindian, Mapuche, or Aymara) and the COPD mortality rate (or hospital discharge rate) was determined through a regression model. For this calculation, the statistical power method in multiple regression was used, considering an effect size of 0.35 (a big effect according to Cohen [39], a significance level of 0.05, and a power of 90%. The estimation of the sample size was performed using the pwr.f2.test [40] function of the pwr R package (version 1.3.0), resulting in a size of 32 communes.

We studied the associations between the average proportion of different ancestries per commune and COPD morbimortality through correlation analysis and negative binomial regression models to predict each rate (hospital discharge and specific mortality) according to each ancestry (Amerindian, Mapuche, and Aymara). We chose negative binomial regression models instead of Poisson regression since we found overdispersion. The average ancestry percentages corresponded to the predictor variables, and the hospital discharge and mortality rates to the response variables.

To isolate the effect of ancestry per se, we decided to incorporate variables of recognized influence on morbidity and mortality rates that may or may not be associated with ancestry percentages of the Chilean population. One aspect that has an influence is the socioeconomic level of the population, which is known to be related to the percentage of ancestry [31,41].

Additionally, another relevant factor is the age structure of the population. Thus, we incorporated the following adjustment variables into the negative binomial regression models (Appendix A Table A1 details these adjustment variables at the communal level):

- Communal Development Index (CDI): Calculated in 2020 by the Universidad Autónoma, it corresponds to the multidimensional evaluation of Chile’s communes in economic, educational, and health aspects, taking values ranging from 0 (minimum development) to 1 (maximum development) [42].
- Elderly People Proportion (EPP): Quotient between the number of people over 60 in a commune and the total population. We calculated it from the CENSUS 2017 database [38].
- Smoking rate of 100 or more cigarettes (SMK100): Estimates the proportion of people who have smoked 100 or more cigarettes in their lifetime. This variable was obtained from the National Health Survey (NHS) from years 2016–2017 [43]. We calculated the percentage of people in each commune who have smoked 100 cigarettes or more in their lifetime.

We collected all the information in a Microsoft Excel database and performed all the statistical analyses with R 4.3.1.

This project was reviewed by the Human Research Ethics Committee of the Faculty of Medicine of the University of Chile and approved on 5 July 2022. As this work used anonymous secondary databases of public access (DEIS) and anonymous results grouped by region and commune, we did not require access to volunteers or to carry out a process of informed consent.

## 3. Results

**Figure 1:**
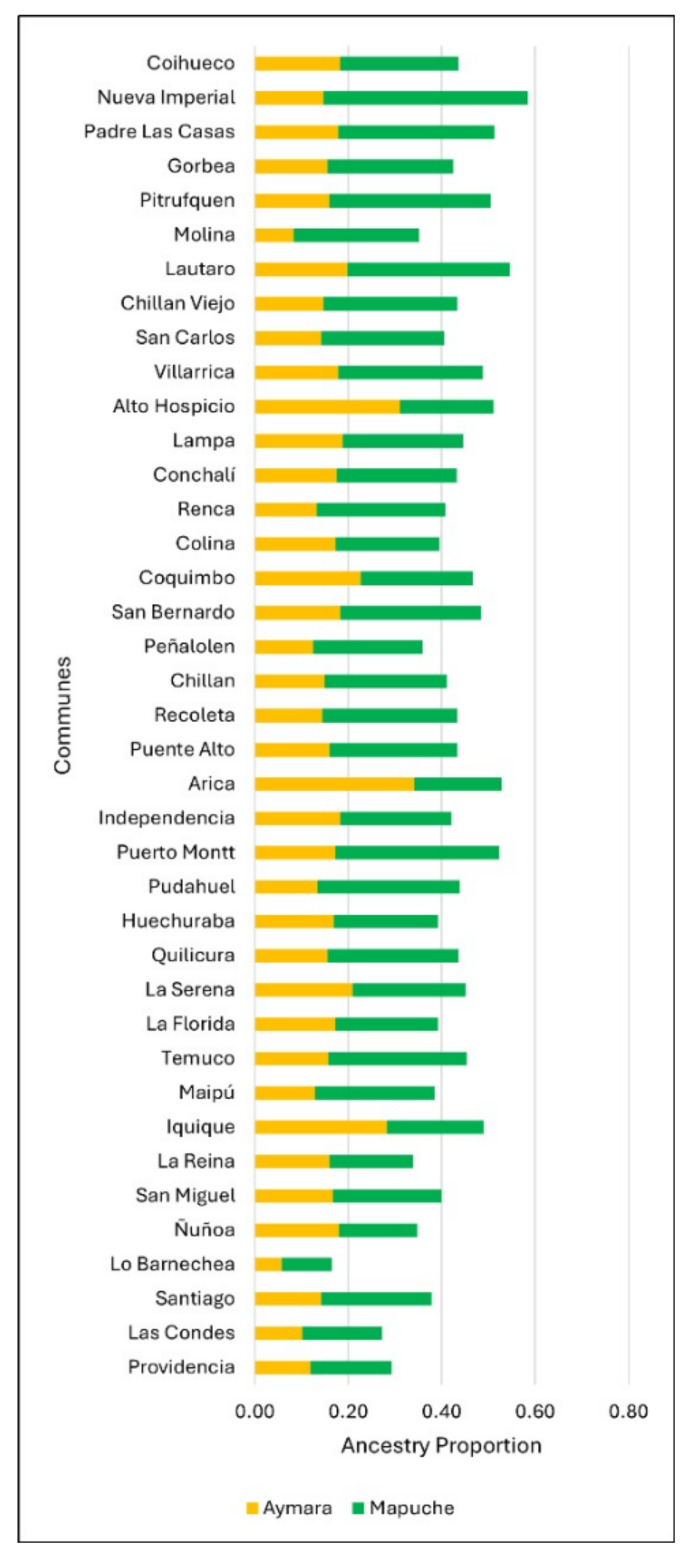
The proportion of Amerindian genomic ancestry by the commune according to origin (Mapuche or Aymara).

The average proportion of Amerindian, Mapuche, and Aymara ancestry was studied in 39 communes. The average Amerindian ancestry varied between 15.1% and 57.1%, and those of specific Mapuche and Aymara ancestry ranged between 7.0% and 43.8% and 5.0% and 34.1%, respectively (Figure 1, Appendix A Table A1).

**Figure 2:**
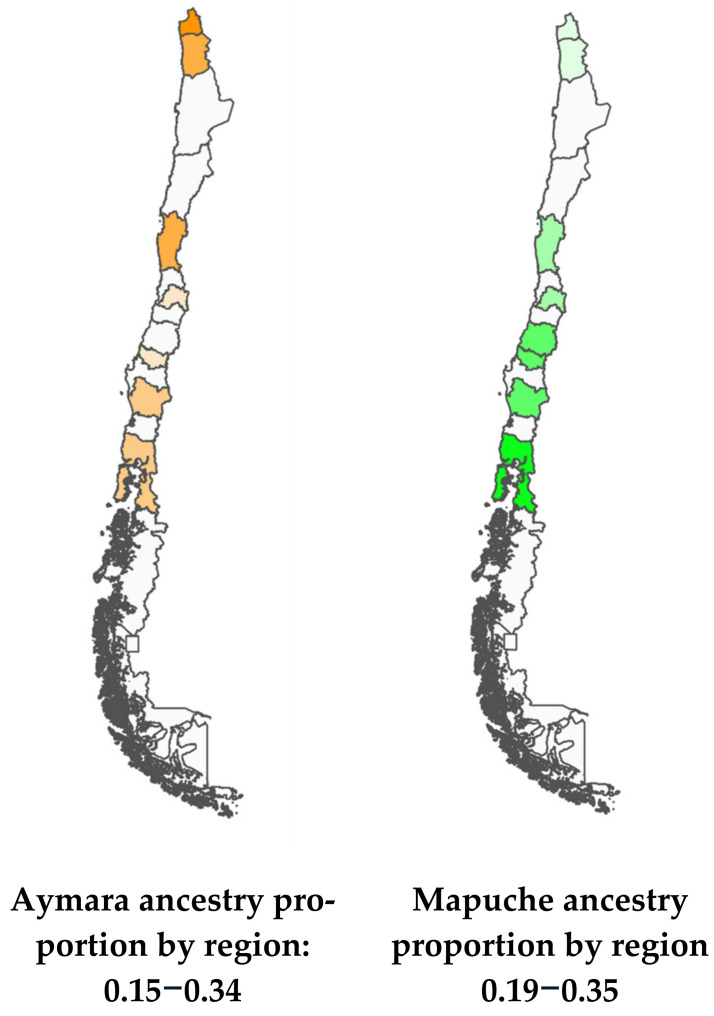
Maps of Chile with Aymara and Mapuche ancestry gradients.

Figure 2 shows the proportions of Mapuche and Aymara ancestry by Region. Nueva Imperial in the South and Arica in the North have the highest percentages of Mapuche and Aymara ancestry, respectively (Figure 2).

The hospital discharges and the regional mortality rates due to COPD were estimated based on the communes included in the study, weighing the communal rates by population size. Higher hospital discharges and mortality rates were observed in the southern regions of the country (Table 1).

Table 2 shows the results of the simple negative binomial regressions considering the averages of the percentages of communal ancestry as the predictor variable and the hospital discharge and mortality rates as the response variable. The last two columns of the table describe the results of the multiple negative binomial regressions, in which the adjustment variables communal development index (CDI), smoking (SMK100), and proportion of elderly population (EPP) were added (these were based on only 36 communes as four did not have information available for SMK100).

**Figure 3:**
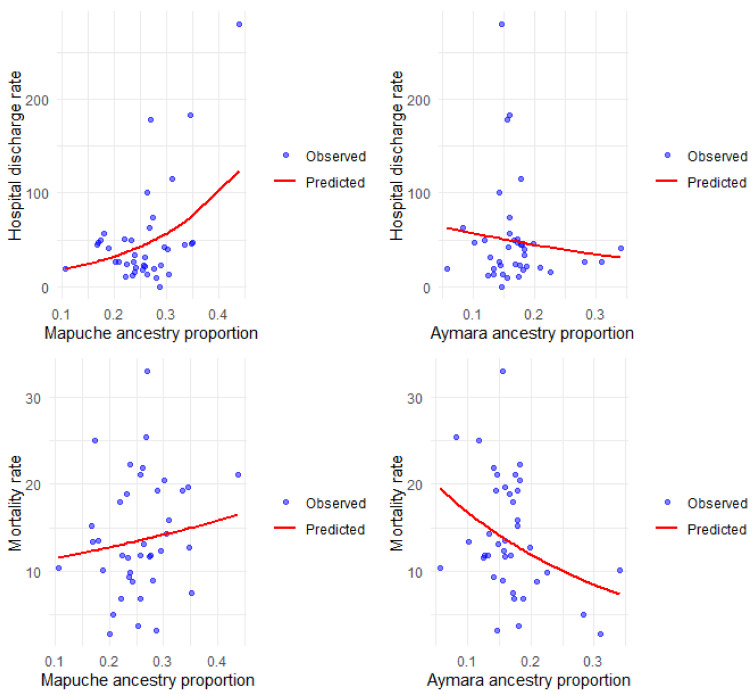
Hospital discharge and mortality rates for COPD per 100,000 inhabitants, observed and predicted, according to Mapuche and Aymara ancestry.

Figure 3 shows the observed and predicted rates according to the simple models, and the details of the multiple regression are described in Table 3.

Regarding the adjustment variables in the multiple models (Table 3), in general, there is a positive relationship between hospital discharge and mortality rates and the proportion of older adults in each commune. The CDI presents an inverse association with hospital discharge rates that is only significant in the model with Aymara ancestry. Furthermore, it is also negatively associated with mortality rates in the model with Amerindian and Aymara ancestry. As for the relationship between tobacco consumption and mortality rates, we find that it is positively and significantly associated with the Amerindian and Mapuche ancestry.

We find a positive and significant association between Mapuche ancestry and hospital discharge rates for COPD, both in the simple and multiple analyses. The value of the incidence rate ratios (IRR) indicates that the higher the Mapuche ancestry in the commune, the higher the rate of hospital discharges for this disease. Aymara ancestry is not significantly associated with the rate of hospital discharges but shows the opposite trend to that observed with Mapuche ancestry. The COPD mortality rate was inversely associated with Aymara ancestry, both in the simple and multiple analysis, indicating that the higher the Aymara ancestry, the lower the COPD mortality rate; the opposite occurred with the percentage of Mapuche ancestry, which was significantly and positively associated with the COPD mortality rate.

## 4. Discussion

In this investigation, we assessed the correlation between the percentage of genomic ancestry of Amerindian origin and the rates of mortality and hospital discharges associated with chronic obstructive pulmonary disease (COPD) within the urban Chilean population across 39 communes. We also analyzed the influence of external variables, including communal development, smoking prevalence, and the percentage of elderly residents.

Our results indicate a significant positive association between Mapuche ancestry and hospital discharges and mortality rates due to COPD. In contrast, Aymara ancestry exhibited a significant inverse relationship, suggesting that it is a protective factor against mortality associated with COPD. This finding implies that the risk factor of Amerindian ancestry differs depending on the specific group, with Mapuche ancestry increasing the risk and Aymara ancestry conferring a degree of protection.

The association observed with genomic ancestry may suggest a potential involvement of genetic risk factors in the pathophysiology of COPD, as the observed differences among various communes reflect the variable magnitudes of Amerindian genomic contributions. Such genetic risk factors could be alleles conferring susceptibility or protection to COPD, with differing frequencies across Chilean subpopulations based on their variable percentages of Amerindian genomic components. The association detected can also be explained by environmental factors and their interaction with the genotype. Many environmental and social factors influence COPD predisposition, such as air pollution, smoking habits, access to healthcare, healthy behavior, and socioeconomic level, among others.

It is known that ancestry is intrinsically linked to the sociocultural context of these communities. The fact that this study focused on a quantitative and continuous gradient of genomic ancestry percentages rather than a comparison between discrete groups of different cultures belonging to some group and that we specifically examined urban populations that self-identified as Chilean (not members of a particular indigenous group and living in rural areas), contributes to consider a diminished impact of social factors, but not discard them.

It is important to remember that the contemporary Chilean mixed population has two relevant ancestral components: European and Amerindian. This study is based on estimates of the percentages of ancestry of the Chilean urban population whose parents are Chilean, excluding recent immigrants from other South American countries. These groups were still a minority in most of the country’s communes at the time the data we used was collected.

Our findings indicate that there would be a higher susceptibility to suffer from or die of COPD the higher the Mapuche ancestry of the individual, while the opposite would happen as the percentage of the genome from northern Amerindian (mainly Aymara) ancestry increases. This fact could explain the findings described in a Chilean study that aimed to associate 80 risk SNPs for COPD, already detected in the Caucasian population, in a sample of a mixed Chilean population [21]. The study revealed that only variants in two genes previously reported in Caucasians demonstrated any degree of association with COPD in the Chilean sample, and this was observed exclusively in a subset of the Chilean population characterized by reduced Mapuche ancestry. The absence of genome-wide association studies (GWAS) in South American populations represents a significant gap in our understanding, as such studies could uncover novel protective or risk alleles for COPD that remain unidentified.

In our multivariate analysis, we confirmed the well-established positive correlation between smoking and the prevalence of COPD, as well as the proportion of elderly individuals within various communes, as has been reported [44]. Regarding the adjustment variables considered in our work, we observed an increase in CDI in the eastern communes of Santiago, such as Lo Barnechea, Providencia, and Las Condes. At the same time, it decreased in other regions of the country. This information reflects, to a certain degree, the structural centralization of Chile in terms of development. These specific communes exhibit a higher prevalence of smoking (SMK100), which may suggest an interrelation between CDI and smoking behaviors. Furthermore, it was noted that communes with elevated CDI levels experienced fewer hospitalizations and lower mortality rates due to COPD compared to those with diminished CDI. This observation implies that improved access to healthcare services is a critical determinant in mitigating morbidity and mortality associated with this disease. As in other studies, we found that Amerindian ancestry is negatively associated with CDI.

An important limitation of our study is the insufficient availability of comprehensive data to thoroughly analyze critical variables related to respiratory diseases, particularly air pollution. Air quality data remains scarce in Chile and exhibits inconsistencies over time, as recorded by the Servicio de Información Nacional de la Calidad del Aire (SINCA). Consequently, our efforts to aggregate the limited information revealed that only a few communes possess data on annual averages of atmospheric pollutants across different years. This is regrettable, as both air quality and smoking are significantly implicated in the pathophysiology of COPD. Our study suffers from another limitation in the analysis of morbidity, as there is an absence of prevalent studies for COPD in our country. As a substitute, we used hospital discharge rates, which, even though they are correlated with the prevalence rate, are affected by healthcare access disparities and personal habits around seeking medical attention. The analysis of mortality rates, on the other hand, is much less debatable.

Despite the above, this study contributes new evidence that holds considerable epidemiological significance, providing a foundation for future inquiries into this important area of research.

Further studies that could incorporate more relevant variables are needed to verify our findings. The confirmation of our found association would suggest that genomic regions of Mapuche origin in Chilean mestizos with COPD may be enriched with risk alleles for the disease, and genomic regions of Aymara origin could harbor protective alleles. Both aspects would help detect new polygenes linked to COPD, which could explain part of the unexplained heritability.

## 5. Conclusions

The present study provides evidence of a significant association between the prevalence and mortality of COPD and Mapuche ancestry in Chilean communes, suggesting that this genomic ancestry is a risk factor for the disease. This relationship persists even when accounting for socioeconomic conditions and the demographic distribution of older individuals within the studied communes. These findings imply that predisposition to and mortality from COPD increases together with the percentage of Mapuche genomic ancestry in Chilean mestizos. The finding that Aymara genomic ancestry has an opposite effect is particularly relevant, as it suggests that considering Amerindian ancestry as a homogeneous entity may obscure specific risk factors. This, in turn, could hinder the identification of relevant etiological determinants in disease research. Additionally, these findings imply that COPD prevention policies should focus on Chilean subpopulations with higher Mapuche ancestry.

Among the confounding variables analyzed, the proportion of the population over 60 years and the higher CDI emerged as risk and protective factors, respectively, reinforcing previously recognized trends in COPD epidemiology [45].

## Figures and Tables

**Table 1:** Hospital discharge and specific mortality rates by Region per 100,000 inhabitants (HDR = hospital discharge rate; SMR = specific mortality rate).

Region	COPD	
	HDR	SMR
Arica	40.83	9.97
Tarapacá y Parinacota	26.69	4.20
Coquimbo	17.67	9.30
Metropolitana	36.47	13.86
Maule	62.46	25.50
Ñuble	27.68	12.77
La Araucanía	75.60	15.15
Los Lagos	46.78	7.47

**Table 2:** Results of simple and multiple negative binomial regressions predicting hospital discharges and mortality rates due to COPD as a function of ancestry percentages.

Tasa	Ancestry-	r	IRR	*p*-Value	adj. IRR	adj. *p* Value
Discharges	Amerindian	0.354	34.347	0.025	26.958	0.050
	Mapuche	0.503	277.023	0.004	103.750	0.024
	Aymara	−0.096	0.087	0.333	1.233	0.921
Mortality	Amerindian	−0.061	0.481	0.401	0.947	0.951
	Mapuche	0.194	2.994	0.358	10.252	0.031
	Aymara	−0.359	0.032	0.004	0.104	0.023

r = Pearson correlation coefficient, IRR = incidence rate ratio. Adjusted IRR and *p*-value were obtained from a multiple model adjusting for CDI (communal development index), SMK100 (proportion of people who have smoked 100 or more cigarettes), and proportion of older adults (EPP).

**Table 3:** Results of multiple negative binomial regressions for predicting hospital discharge and mortality rates for COPD as a function of ancestry, communal development index (CDI), smoking (SMK100), and elderly people proportion (EPP) in the commune.

Tasa	Predictor Variables	Amerindian		Mapuche		Aymara	
		IRR	*p*	IRR	*p*	IRR	*p*
Discharges	Ancestry	26.958	0.050	103.750	0.024	1.233	0.921
	CDI	0.531	0.537	0.850	0.878	0.145	<0.001
	SMK100	1.438	0.634	0.828	0.799	1.244	0.020
	EPP	465,162.100	<0.001	981,118.9	<0.001	308,832.000	<0.001
Mortality	Ancestry	0.947	0.951	10.252	0.031	0.104	0.023
	CDI	0.312	0.031	0.570	0.282	0.285	0.005
	SMK100	2.630	0.013	2.494	0.011	2.116	0.050
	EPP	6920.118	<0.001	12,434.110	<0.001	4673.275	<0.001

## Data Availability

The data presented in this study are available on GitHub at https://github.com/andreacanals/COPD_and_ancestry. These data were derived from the following resources available in the public domain: https://deis.minsal.cl/, http://resultados.censo2017.cl, http://genoma.med.uchile.cl/ancestry, https://epi.minsal.cl/encuesta-nacional-de-salud-2015-2016, https://ediciones.uautonoma.cl/index.php/UA/catalog/download/6/11/22?inline=1 (all accessed on 31 January 2024).

## Abbreviations

The following abbreviations are used in this manuscript:

AIMs	Ancestry Informative Markers
COPD	Chronic Obstructive Pulmonary Disease
DEIS	Departamento de Estadísticas e Información en Salud
GWAS	Genome-Wide Association Studies
CDI	Communal Development Index
EPP	Elderly People Proportion
SMK100	Proportion of people who have smoked 100 or more cigarettes
r	Pearson correlation coefficient
IRR	Incidence rate ratio
HD	Hospital discharge ratio
SMR	Specific mortality ratio

## Appendix A

**Table A1:** Percentages of Aymara and Mapuche ancestry and adjustment variables by commune.

Commune	%AYM	%MAP	EPP	CDI	SMK100
Arica	0.34	0.18	0.15	0.54	0.29
Iquique	0.28	0.20	0.15	0.60	0.48
Alto Hospicio	0.31	0.20	0.07	0.41	0.30
La Serena	0.20	0.24	0.15	0.58	0.53
Coquimbo	0.22	0.23	0.14	0.52	0.36
Colina	0.17	0.22	0.09	0.52	0.57
Lampa	0.18	0.25	0.09	0.45	0.55
Lo Barnechea	0.05	0.10	0.11	0.67	0.67
Quilicura	0.15	0.28	0.09	0.58	0.52
Huechuraba	0.16	0.22	0.12	0.56	0.20
Conchalí	0.17	0.25	0.18	0.47	0.60
Renca	0.13	0.27	0.14	0.48	0.50
Recoleta	0.14	0.28	0.17	0.54	0.38
Independencia	0.18	0.23	0.15	0.54	0.78
Las Condes	0.10	0.16	0.20	0.88	0.75
Pudahuel	0.13	0.30	0.12	0.55	0.40
Providencia	0.11	0.17	0.20	0.88	0.71
Santiago	0.14	0.23	0.11	0.78	0.66
La Reina	0.16	0.17	0.19	0.61	0.56
Ñuñoa	0.17	0.16	0.19	0.66	0.42
San Miguel	0.16	0.23	0.17	0.61	0.69
Peñalolen	0.12	0.23	0.14	0.53	0.53
Maipu	0.12	0.25	0.13	0.60	0.51
La Florida	0.17	0.21	0.17	0.59	0.38
San Bernardo	0.18	0.30	0.12	0.52	0.74
Puente Alto	0.16	0.27	0.11	0.54	0.53
Molina	0.08	0.26	0.17	0.37	0.50
Coihueco	0.18	0.25	0.15	0.30	0.26
San Carlios	0.14	0.26	0.18	0.41	0.30
Chillán	0.14	0.26	0.16	0.54	0.40
Chillán Viejo	0.14	0.28	0.13	0.38	0.54
Lautaro	0.19	0.34	0.15	0.37	
Temuco	0.15	0.29	0.14	0.60	0.45
Nueva Imperial	0.14	0.43	0.18	0.31	0.50
Padre Las Casas	0.17	0.33	0.13	0.32	0.24
Pitrufquen	0.15	0.34	0.19	0.35	
Gorbea	0.15	0.26	0.21	0.33	
Villarica	0.17	0.31	0.16	0.41	0.27
Puerto Montt	0.17	0.35	0.12	0.54	0.35

[CDI=Communal Development Index; SMK100: rate of people who smoked 100 or more cigarettes; EPP: Elderly People Proportion; %MAP: Mapuche ancestry proportion; %AYM: Aymara ancestry proportion.] (Unable data were left blank).
